# Nine- to Twelve-Month Anti-Tuberculosis Treatment Is Associated with a Lower Recurrence Rate than 6–9-Month Treatment in Human Immunodeficiency Virus-Infected Patients: A Retrospective Population-Based Cohort Study in Taiwan

**DOI:** 10.1371/journal.pone.0144136

**Published:** 2015-12-03

**Authors:** Jann-Yuan Wang, Hsin-Yun Sun, Jann-Tay Wang, Chien-Ching Hung, Ming-Chih Yu, Chih-Hsin Lee, Li-Na Lee

**Affiliations:** 1 Department of Internal Medicine, National Taiwan University Hospital and National Taiwan University College of Medicine, Taipei, Taiwan; 2 Division of Pulmonary Medicine, Department of Internal Medicine, Wan Fang Hospital, Taipei Medical University, Taipei, Taiwan; 3 Department of Laboratory Medicine, National Taiwan University Hospital and National Taiwan University College of Medicine, Taipei, Taiwan; 4 School of Medicine, College of Medicine, Taipei Medical University, Taipei, Taiwan; 5 School of Respiratory Therapy, College of Medicine, Taipei Medical University, Taipei, Taiwan; National Taiwan University Hospital, TAIWAN

## Abstract

**Background:**

Human immunodeficiency virus (HIV)-infected patients are at an increased risk of tuberculosis (TB) and its recurrence following completion of anti-TB treatment. We investigated whether extending anti-TB treatment to 9 months or longer reduces TB recurrence.

**Methods:**

HIV-infected patients who were diagnosed with pulmonary TB between 1997 and 2009 and who received anti-TB treatment for a duration between 5.5 and 12.5 months were identified from the National Health Insurance Research Database in Taiwan. Those who received any non-fluoroquinolone second-line anti-TB drug for >28 days were excluded. Factors associated with TB recurrence within 2 years after completion of anti-TB treatment were explored using Cox regression analysis. Sensitivity analysis was performed for a subpopulation fulfilling strict diagnostic criteria for HIV infection.

**Results:**

TB recurrence was observed in 18 (3.5%) of 508 HIV-infected patients. The recurrence rate declined from 5.4% to 1.0% after the implementation of directly observed therapy, short course (DOTS) in 2006 (*p* = 0.014). The recurrence rate was 5.9%, 5.2%, and 1.6% in patients who received anti-TB treatment for <195, 195–270, and >270 days, respectively (*p* = 0.066). Cox regression analysis revealed that TB diagnosed in the DOTS era (hazard ratio [HR]: 0.18 [0.04–0.77]) and anti-TB treatment for >270 days (HR: 0.24 [0.06–0.89]) were associated with a reduced risk of TB recurrence. Sensitivity analysis of 449 selected patients revealed that anti-TB treatment for >270 days was a significant factor.

**Conclusion:**

In Taiwan, the 2-year TB recurrence rate in HIV-infected patients declined after implementation of DOTS. The risk of TB recurrence in HIV-infected patients can be further reduced by extending anti-TB treatment to 9–12.5 months.

## Introduction

Tuberculosis (TB) is a global public health concern. In 2011, an estimated 8.7 million new cases of TB were identified globally [1]. Using the current standard anti-TB treatment, a cure rate of more than 95% can be achieved for pulmonary TB in human immunodeficiency virus (HIV)-negative patients, with a 2-year recurrence rate of 2%–3% [2, 3]. For TB patients co-infected with HIV, however, increased recurrence after completion of anti-TB treatment has been reported [4–7].

Current recommendation for anti-TB regimen and treatment duration is the same for patients with or without HIV [8]. In recent years, new data suggest that the TB recurrence rate of HIV-TB co-infected patients may be reduced by administering rifamycin-based treatment for more than 6 months [5, 7]. A randomized controlled trial finds that HIV-infected patients treated with a 9-month treatment had a lower recurrence rate than those with a 6-month regimen [9]. Nevertheless, the optimal duration of anti-TB therapy in HIV-infected patients remains uncertain.

The National Health Insurance program in Taiwan is a mandatory universal health insurance program that has offered comprehensive medical care coverage to 99% of residents in Taiwan since 1996 [10]. The National Health Insurance Research Database (NHIRD) provides suitable research material for exploring the impact of medical intervention on the outcome of populations with chronic infectious diseases [11]. In this study, we determined the risk factors for TB recurrence in HIV-infected patients by using the NHIRD, with emphasis on the impact of the anti-TB treatment duration.

## Patients and Methods

This study was approved by the Research Ethics Committee of National Taiwan University Hospital, Taipei, Taiwan (NTUH REC: 201112111RIC). Because this was a retrospective study that used an encrypted database, the need for informed consent was waived.

Comprehensive healthcare data in the NHIRD, including enrollment files, claims data, catastrophic illness files, and registry for drug prescriptions, were screened to identify patients with pulmonary TB and HIV coinfection. Their clinical characteristics and medical information were retrieved.

### Patient Selection

**Fig 1:**
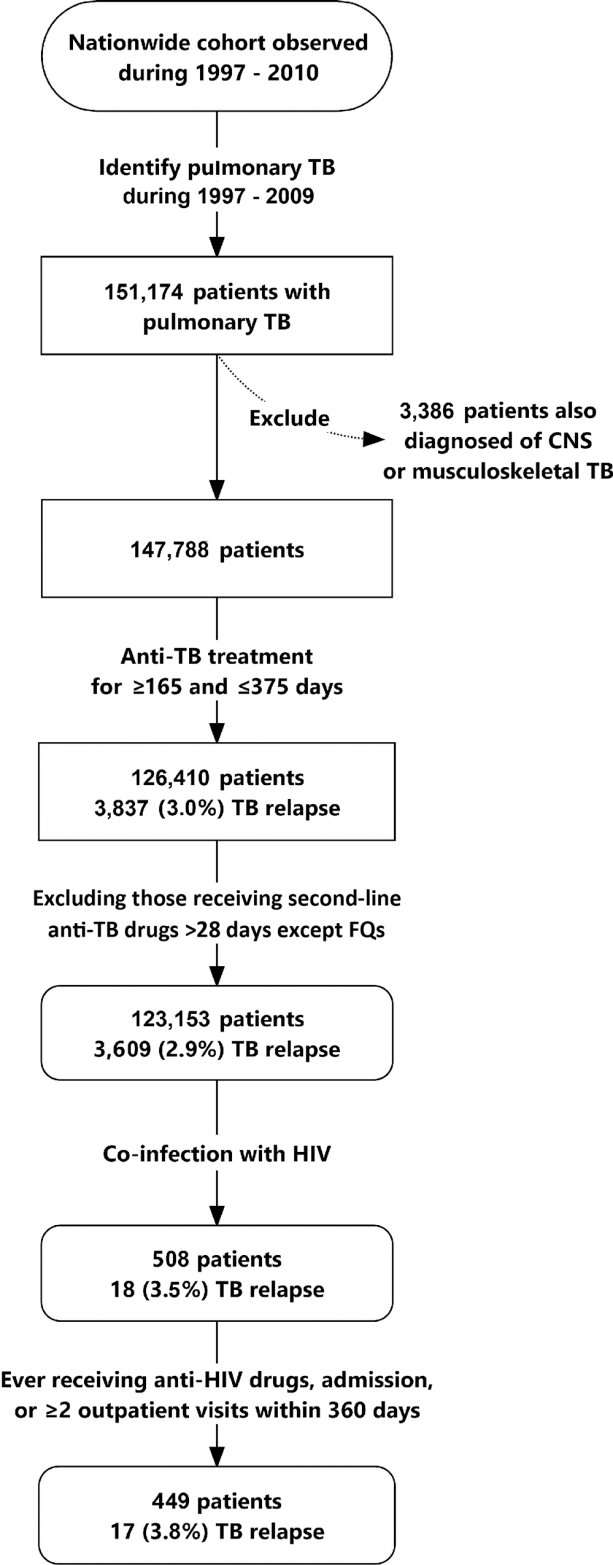
Selection of patients with pulmonary tuberculosis (TB) and human immunodeficiency virus (HIV) infection from the National Health Insurance Research Database in Taiwan.

Patients diagnosed with pulmonary TB from 1997 and 2009 were identified (Fig 1). Because the recommended treatment duration is long, patients with central nervous system (CNS) or musculoskeletal TB were excluded. To prevent the inclusion of patients with drug-resistant TB (particularly multidrug-resistant TB) or adverse reactions due to first-line anti-TB drugs, only patients who received the anti-TB regimen for a duration between 165 and 375 days (5.5–12.5 months) were selected, and those who received any non-fluoroquinolone second-line anti-TB drug for >28 days were excluded. Patients were classified into 3 groups using the treatment durations of 195 and 270 days as the cutoff points: <195 days (<6.5 months), 195–270 days (6.5–9 months), and >270 days (>9 months).

Underlying comorbidities were recorded if they were present before the diagnosis of pulmonary TB, according to a previous publication [12]. The low-income group was defined as having an annual household income <4500 US dollars according to enrollment files [13].

The primary outcome was time to TB recurrence within 2 years after completion of anti-TB treatment for the first episode of TB [1]. Patients were followed up for 2 years after completion of anti-TB treatment until TB recurrence; December 31, 2010; or withdrawal from the health insurance program.

### Definition of Active Pulmonary Tuberculosis

Patients with active pulmonary TB were identified as those who had at least 2 ambulatory visits or 1 inpatient record with a compatible diagnosis, plus at least 1 prescription of 3 or more anti-TB drugs and prescriptions of at least 2 anti-TB drugs simultaneously for ≥120 days within a period of 180 days (see S1 File for details) [14]. Among them, those diagnosed with CNS or musculoskeletal TB were excluded. Data on individual anti-TB agents were extracted from the claims data and converted to the defined daily doses (DDDs), and the agents were grouped according to their pharmacologic categories [15]. The prescriptions of patients with end-stage renal disease were adjusted according to treatment guidelines [16].

The date of completion of anti-TB treatment was defined as the final date of the simultaneous intake of 2 or more anti-TB drugs without further anti-TB treatment in the next 60 days. Anti-TB treatment was considered 80% consistent with the standard regimen if it consisted of isoniazid (INH), a rifamycin, ethambutol (EMB), and pyrazinamide (PZA) for >48 days in the first 2 months and INH and a rifamycin for >144 days in the first 6 months. Directly observed therapy, short course (DOTS) has been implemented in Taiwan since 2006.

### Definition of Tuberculosis Recurrence

Because HIV-infected patients with TB recurrence might die before or during anti-TB treatment, TB recurrence was defined as the following conditions: (1) a recurrent episode of active pulmonary TB after completion of treatment, (2) a positive culture for *Mycobacterium tuberculosis* and death or loss to follow-up in the next 3 months, and (3) simultaneous use of 3 anti-TB drugs within the final 3 months before death or loss to follow-up. Because no culture results are available in the NHIRD, the drug susceptibility test for *M*. *tuberculosis* was used as a proxy for positive TB cultures.

### Definition of Human Immunodeficiency Virus Infection and Combination Antiretroviral Therapy

TB patients with a diagnostic code for HIV infection within 3 years were considered to have HIV coinfection (see S1 File). Combination antiretroviral therapy (cART), which became available in Taiwan in early 1997, was defined as the simultaneous use of 2 nucleoside reverse-transcriptase inhibitors (NRTIs) and a non-NRTI (NNRTI) or a protease inhibitor (PI) [17, 18]. Anti-HIV treatment that did not fulfill the definition of cART was denoted as “anti-HIV treatment but not cART.”

### Statistical Analysis

Intergroup differences were calculated using one-way analysis of variance (ANOVA) for continuous variables and the chi-square test for categorical variables, as appropriate. Cox proportional hazards regression analysis was performed to evaluate the impacts of age, sex, comorbidities, income status, anti-HIV treatment, timing of TB diagnosis, and duration of anti-TB treatment on the risk of TB recurrence. Proportionality was tested by including time-dependent covariates in the Cox model. The entry and stay levels in the stepwise variable selection procedure were set at 0.15. Only variables with a 2-sided *p* < 0.05 were included in the final model. Model fitting was measured by using −2 log likelihood. All analyses were performed using SAS version 9.2 (SAS Institute Inc., Cary, NC, USA).

### Sensitivity Analysis

Sensitivity analysis was performed to verify statistical findings in a subpopulation fulfilling strict diagnostic criteria for HIV infection (Fig 1). Patients were selected if they had received cART, had at least 1 admission with the discharge diagnosis of HIV infection, and had 2 outpatient visits with the diagnosis of HIV within 360 calendar days.

## Results

**Fig 2:**
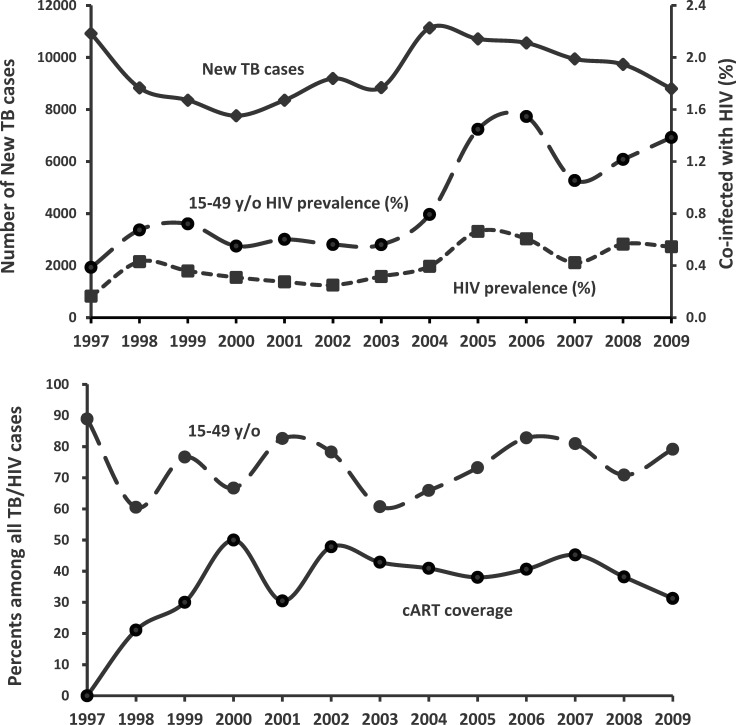
Number of annual cases among the 123,153 identified pulmonary tuberculosis (TB) cases and percentages of patients living with human immunodeficiency virus (HIV) in all age groups and in those aged between 15 and 49 years (upper panel), and percentages of patients aged between 15 and 49 years and those receiving combination antiretroviral therapy (cART) during anti-TB treatment among all patients with TB–HIV coinfection (lower panel).

A total of 151,174 patients were diagnosed with pulmonary TB between 1997 and 2009 (Fig 1). Among them, 126,410 did not have CNS or musculoskeletal TB and received anti-TB treatment for a duration ranging from 165 to 375 days. Among the 126,410 patients, 3,257 were excluded because they received non-fluoroquinolone second-line anti-TB drugs for more than 28 days. Among the 123,153 patients, 508 (0.34%) had HIV coinfection. The number of cases was the highest in 2004 (11,136) and the lowest in 2009 (8,802), whereas HIV seroprevalence was the lowest in 1997 (0.16%) and the highest in 2005 (0.66%) (Fig 2A). HIV seroprevalence in patients aged between 15 and 49 years (377 in 42,911, 0.88%) was significantly higher than that in other age groups (131 in 80,240, 0.16%) (*p* < 0.001) and was the highest in 2006 (1.55%). The coverage rate of cART among HIV-infected patients increased gradually from 0% in 1997 to the highest rate of 50.0% in 2000 and remained above 30% thereafter (Fig 2B).

**Fig 3:**
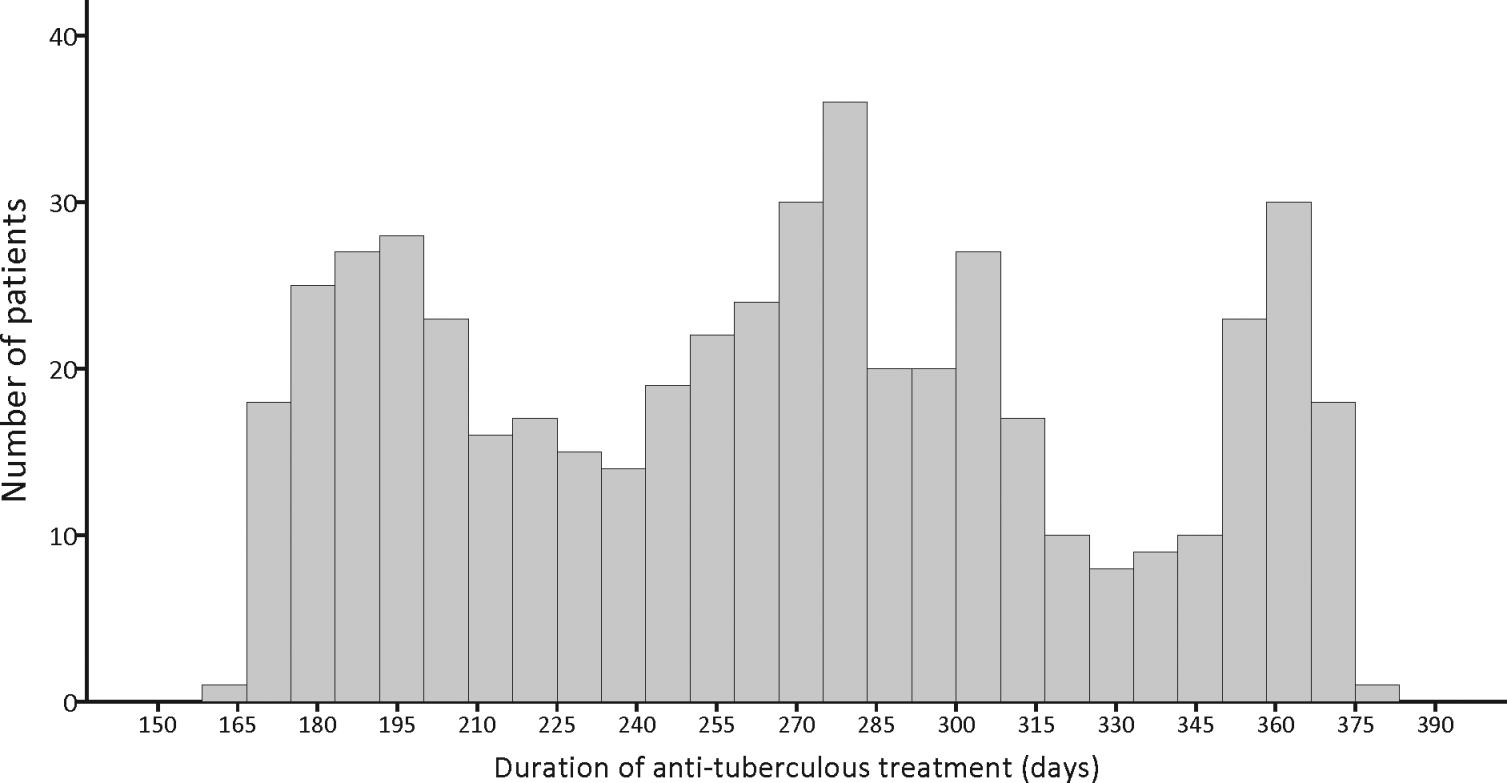
Histogram of duration of anti-tuberculosis treatment of the 508 patients.

The clinical characteristics of the 508 patients with TB and HIV coinfection are summarized in Table 1. The mean age was 42.4 ± 14.8 years, and 89.4% were men. A histogram of the duration of anti-TB treatment is shown in Fig 3. The treatment duration was <195 days (<6.5 months) in 85 (16.7%) and >270 in 249 patients (49.0%). During anti-TB treatment, 185 (36.4%) received cART. Anti-TB treatment was more than 80% consistent with the standard regimen in only 185 patients (36.4%). Among 10,315.1 person-months of follow-up, TB recurrence was observed in 18 (3.5%) of the 508 patients within 2 years after completion of anti-TB treatment. The recurrence rate declined from 5.4% (16 in 299) before DOTS implementation (1997–2005) to 1.0% (2 in 209) after (2006–2009) DOTS implementation.

**Table 1:** Clinical characteristics of the 508 patients with human immunodeficiency virus infection.

	<195 days (n = 85)	195–270 days (n = 174)	>270 days (n = 249)
Age (years)	41.5 ± 15.1	42.6 ± 15.2	42.5 ± 14.4
Age ≥50	18 (21.2%)	46 (26.4%)	67 (26.9%)
Male	71 (83.5%)	159 (91.4%)	224 (90.0%)
Timing of TB diagnosis			
Pre-DOTS era	46 (54.1%)	101 (58.0%)	152 (61.0%)
DOTS era	39 (45.9%)	73 (42.0%)	97 (39.0%)
Comorbidity	11 (12.9%)	22 (12.6%)	27 (10.8%)
Diabetic mellitus	7 (8.2%)	15 (8.6%)	23 (9.2%)
Chronic obstructive pulmonary disease	3 (3.5%)	3 (1.7%)	3 (1.2%)
Malignancy*	3 (3.5%)	5 (2.9%)	1 (0.4%)
End-stage renal disease	0 (0.0%)	1 (0.6%)	1 (0.4%)
Low income	3 (3.5%)	5 (2.9%)	6 (2.4%)
Duration of anti-TB treatment (days)**	182.2 ± 8.3	233.5 ± 23.7	317.3 ± 34.1
Total duration of isoniazid administration**	152.7 ± 49.9	178.6 ± 77.0	233.2 ± 110.7
≥240 days**	0 (0.0%)	33 (19.0%)	173 (69.5%)
Total duration of rifamycin administration**	155.6 ± 38.8	181.5 ± 60.6	237.0 ± 91.2
≥240 days**	0 (0.0%)	23 (13.2%)	156 (62.7%)
Total duration of ethambutol administration**	145.1 ± 42.4	180.1 ± 57.6	248.5 ± 81.0
≥240 days**	0 (0.0%)	21 (12.1%)	177 (71.1%)
Total duration of pyrazinamide administration*	80.4 ± 46.5	89.4 ± 69.2	106.0 ± 89.7
≥60 days	59 (69.4%)	107 (61.5%)	162 (65.1%)
≥90 days	27 (31.8%)	66 (37.9%)	103 (41.4%)
80% consistent with standards** ^#^	37 (43.5%)	46 (26.4%)	51 (20.5%)
Intensive phase (initial 60 days)			
No. of days of isoniazid administration*	52.0 ± 16.5	46.9 ± 19.1	44.6 ± 20.7
No. of days of rifamycin administration**	52.7 ± 10.3	46.6 ± 14.9	45.2 ± 16.6
No. of days of ethambutol administration	53.0 ± 9.6	50.7 ± 10.2	49.8 ± 12.5
No. of days of pyrazinamide administration*	47.6 ± 17.3	40.2 ± 20.3	40.6 ± 19.7
Anti-HIV therapy during anti-TB treatment**			
cART	19 (22.4%)	53 (30.5%)	113 (45.4%)
Yes, but not cART	17 (20.0%)	38 (21.8%)	67 (26.9%)
No	49 (57.6%)	83 (47.7%)	69 (27.7%)
2-year recurrence after anti-TB treatment	5 (5.9%)	9 (5.2%)	4 (1.6%)

cART, combination antiretroviral therapy; DOTS, directly observed therapy, short course; TB, tuberculosis.

Data are presented as numbers (%) or means ± SD.

*p < 0.05 and

**p < 0.001 between the 3 groups with different durations of anti-TB treatment according to one-way analysis of variance for continuous variables or the chi-square test for categorical variables.

^#^receiving isoniazid, rifamycin, ethambutol, and pyrazinamide for >48 days in the first 2 months, and isoniazid and rifamycin for >144 days in the first 6 months of anti-TB treatment.

The 2-year recurrence rate was 5.9%, 5.2%, and 1.6% in patients who received anti-TB treatment for <195 days, 195–270 days, and >270 days, respectively (*p* = 0.066 by one-way ANOVA) (Table 1). Significant differences were observed in the duration of anti-TB treatment and total exposure duration of each anti-TB drug among the 3 groups, whereas no significant differences were observed in age, sex, underlying comorbidities, except malignancy, and income status among the 3 groups. The proportions of patients receiving PZA for ≥90 days were 31.8%, 37.9%, and 41.4% (*p* = 0.285 by one-way ANOVA). Significant differences were observed in the proportion of patients receiving anti-TB treatment that was 80% consistent with the standard regimen (*p* < 0.001). The exposure duration of each anti-TB drug among the 134 adherent cases was significantly longer than that among the 374 non-adherent cases (S1 Table). In the latter group, an insufficient duration of rifamycin was more common than that of INH (S2 Table). Significant differences were observed in the initiation of cART among the 3 groups with different duration of anti-TB treatment (22.4% vs. 30.5% vs. 45.4%, *p* < 0.001). The exposure duration of rifamycin, but not other anti-TB drugs, was significantly different among the patients with different status of anti-HIV therapy (S3 Table).

**Fig 4:**
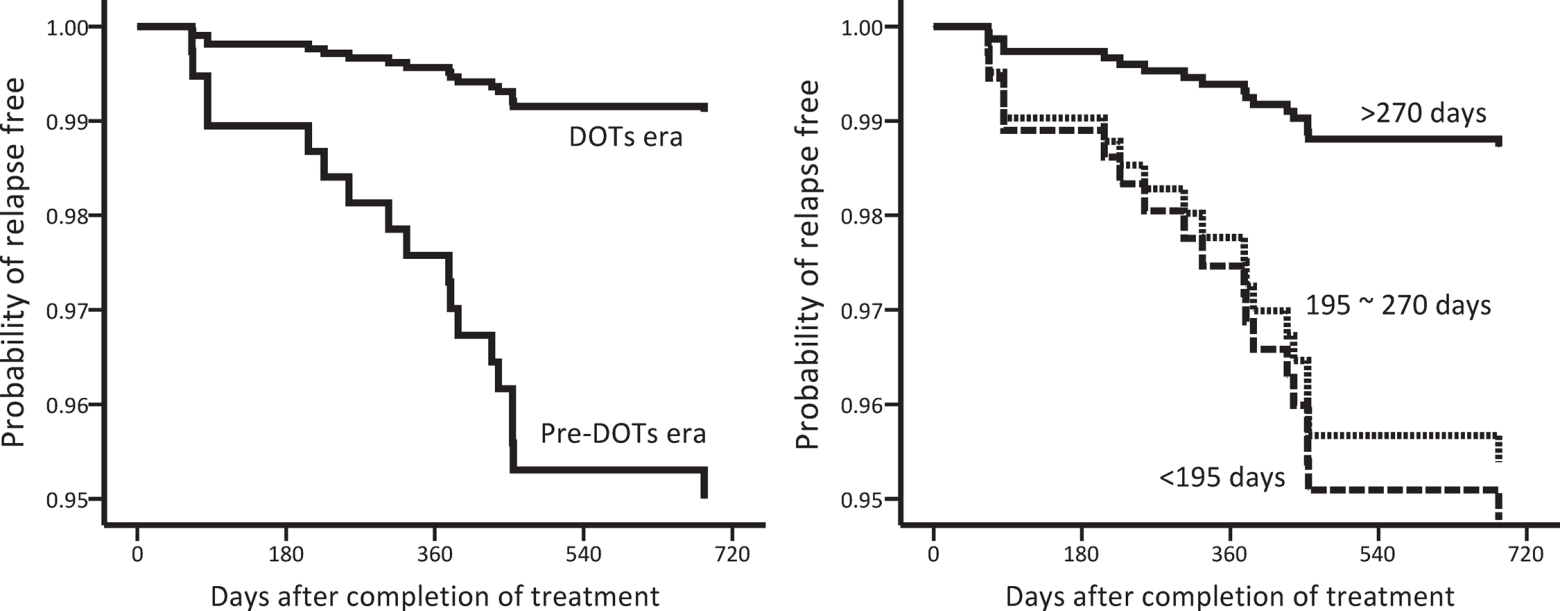
Adjusted time-to-recurrence curves for the 508 patients with human immunodeficiency virus infection stratified by the timing of tuberculosis (TB) diagnosis (left panel) and duration of anti-TB treatment (right panel) were plotted on the basis of regression estimates in the Cox model and average covariate values (average covariate method).

Table 2 shows the risk and timing of TB recurrence in different subgroups and a comparison of the groups according to the log-rank test. Factors associated with a lower risk of TB recurrence were TB diagnosed in the DOTS era (*p* = 0.014), anti-HIV therapy but not cART during anti-TB treatment (*p* = 0.042), anti-TB treatment for >270 days (*p* = 0.056), and total duration of INH use ≥240 days (*p* = 0.030), and duration of rifamycin use ≥240 days (*p* = 0.090). Multivariate Cox regression analysis revealed that TB diagnosed in the DOTS era (hazard ratio [HR]: 0.18 [0.04–0.77]) and anti-TB treatment for >270 days (HR: 0.24 [0.06–0.89]) were independently associated with a low risk of TB recurrence within 2 years after completion of anti-TB treatment (Table 3 and Fig 4).

**Table 2:** Risk of tuberculosis (TB) recurrence in different subgroups.

Characteristics		No. at risk	No. (%) with recurrence	*p* value*
Age (years)	>49	131	6 (4.6%)	0.410
	≤49	377	12 (3.2%)	
Sex	Women	54	0 (0%)	0.147
	Men	454	18 (4.0%)	
Timing of TB diagnosis	Pre-DOTS era	299	16 (5.4%)	0.014
	DOTS era	209	2 (1.0%)	
Comorbidity	Present	60	1 (1.7%)	0.429
	Absent	448	17 (3.8%)	
Low income	Yes	14	0 (0%)	0.472
	No	494	18 (3.6%)	
Anti-HIV Tx during anti-TB Tx	cART	185	5 (2.7%)	0.042
	Not cART	122	1 (0.8%)	
	No	201	12 (6.0%)	
Duration of anti-TB treatment	<195 days	85	5 (5.9%)	0.056
	195–270 days	174	9 (5.2%)	
	>270 days	249	4 (1.6%)	
Total duration of isoniazid administration	<240 days	302	15 (5.0%)	0.030
	≥240 days	206	3 (1.5%)	
Total duration of rifamycin administration	<240 days	329	15 (4.6%)	0.090
	≥240 days	179	3 (1.7%)	
Total duration of ethambutol administration	<240 days	310	14 (4.5%)	0.109
	≥240 days	198	4 (2.0%)	
Total duration of pyrazinamide administration	<60 days	180	5 (2.8%)	0.410
	≥60 days	328	13 (4.0%)	
Consistency with standards^#^	>80%	134	4 (3.0%)	0.734
	≤80%	374	14 (3.7%)	

cART, combination antiretroviral therapy; DOTS, directly observed therapy, short course; HIV, human immunodeficiency virus; Tx, treatment.

*Timing of TB recurrence was compared using the log-rank test.

^#^receiving isoniazid, rifamycin, ethambutol, and pyrazinamide for >48 days in the first 2 months, and isoniazid and rifamycin for >144 days in the first 6 months of anti-TB treatment.

**Table 3:** Determination of independent risk factors for tuberculosis (TB) recurrence within 2 years after completion of anti-TB treatment among the 508 patients with human immunodeficiency virus infection by using Cox proportional hazards regression analysis.

	*p* value	Hazard ratio	95% CI	
			Lower	Upper
Timing of TB diagnosis: DOTS era vs. pre-DOTS era	0.021	0.18	0.04	0.77
Duration of anti-TB treatment:				
>270 days vs. <195 days	0.033	0.24	0.06	0.89
195–270 days vs. <195 days	0.818	0.88	0.30	2.63

DOTS, directly observed therapy, short course.

Among the 508 patients, 449 were selected for sensitivity analysis (Fig 1). Their clinical characteristics (S4 Table) were similar to those of all 508 patients, except that the 449 patients were more likely to be men (93.1% vs. 89.4%; *p* = 0.043). Multivariate Cox regression analysis revealed that TB diagnosed in the DOTS era (HR: 0.19 [0.04–0.84]) and anti-TB treatment for >270 days (HR: 0.25 [0.07–0.87]) were independently associated with a lower 2-year recurrence rate (S5 Table, S1 Fig).

## Discussion

This study demonstrated 2 main findings by using a nationwide cohort in Taiwan. First, inconsistent with a previous report [9], extending the duration of anti-TB treatment to 9–12.5 months was associated with a low 2-year recurrence rate. This benefit was also observed for the subpopulation fulfilling strict diagnostic criteria for HIV infection. Second, the 2-year TB recurrence rate was significantly reduced in TB patients living with HIV in Taiwan after implementation of DOTS. This improvement cannot be explained by the implementation of cART.

Studies have reported that approximately 55%–71% of patients with smear-positive TB who receive the standard 4-drug anti-TB treatment for 2 months exhibit sputum culture conversion [19–21]. The main reason for continuing anti-TB treatment for at least 4 additional months (consolidation phase) is to eradicate residual TB bacilli and prevent recurrence. Although the study design and definition of TB recurrence vary, several studies have demonstrated a recurrence rate of approximately 3.0%–9.3% in HIV-infected patients, which is significantly higher than that in patients without HIV infection or an unknown HIV status (0.8%–5.3%) [4–7]. The reason for the increased TB recurrence rate in HIV-infected patients is multifactorial. First, adverse drug reactions are common in HIV-infected TB patients [22] and typically result in treatment interruption or regimen modification. This study demonstrated that only 26.4% of patients received anti-TB treatment that was 80% consistent with the standard regimen. Second, a low plasma concentration and small area under the concentration–time curve of rifabutin have been noted with coadministration of efavirenz [23] and increase the risk of acquired rifamycin resistance-associated treatment failure and TB recurrence [24]. Third, patients living with HIV have compromised cellular immunity, thus increasing the risk of a recurrent episode of active TB owing to exogenous reinfection.

A meta-analysis showed that prolonging rifamycin treatment from 2–3 months or 5–6 months to ≥7 months led to a significant reduction in the recurrence rate in HIV-infected patients [5]. Furthermore, the beneficial effect of extending the rifamycin treatment duration on TB recurrence was confirmed by 2 randomized clinical trials with head-to-head comparisons of the rifamycin treatment duration; both enrolled approximately 300 HIV-infected TB patients [9, 25]. However, all anti-TB drugs were administered three times weekly throughout the study period in one trial [9], and INH and rifampin were administered twice weekly after the intensive phase in the other trial [25]. Pooled results demonstrated that administration three times weekly during the intensive phase was associated with an 4.8-fold increase in TB recurrence compared with daily administration [26]. In addition to the rifamycin treatment duration, the treatment duration of all anti-TB drugs has been proposed to be a vital predictor of TB recurrence in HIV-infected patients [4, 9].

Our multivariate analysis showed that the overall duration of anti-TB treatment, rather than rifamycin alone, was an independent predictor of recurrence, inconsistent with findings in the literature. This discrepancy may occur because administration of intermittent therapy during the intensive or continuation phase of anti-TB treatment has never been the standard practice in Taiwan [16]. Because this was not a prospective, randomized clinical study, anti-TB treatment may have been extended simply because of a poor therapeutic response. In addition, patients with a higher CD4 count were less likely to be administered cART and more likely to receive a shorter course of anti-TB treatment. Nonetheless, this may not cause major bias because, if that was the case, the 2-year recurrence rate in patients receiving longer anti-TB treatment should not be lower than that in patients receiving standard treatment. Furthermore, the results of sensitivity analysis suggest that treatment prolongation exhibited a benefit in the subpopulation fulfilling strict diagnostic criteria for HIV infection.

Because identifying every possible adverse effect (AE) in the NHIRD is difficult and unreliable, the AEs that developed during extended treatment are unknown. Before extension of anti-TB treatment can be recommended to HIV-infected patients, additional prospective studies should be conducted to clarify its risks, benefits, and cost-effectiveness to facilitate optimal allocation of limited public health and medical resources.

With the implementation of Taiwan’s National TB Program, several action plans have been launched since 2002 to improve TB control [27]. The directly observed therapy (DOT) program with nationwide TB care worker engagement has been provided to every smear-positive case since 2006. In 2007, the DOT program was expanded to include culture-positive cases and special risk groups, including aboriginal populations, uncooperative patients, and the homeless [28]. The findings of this study support continuous government commitment to TB control in Taiwan.

Because of the unavailability of laboratory results and radiologic findings in the claims data, some uncertainty exists regarding the diagnosis of pulmonary TB, HIV infection, therapeutic response, and recurrence. However, this may not be a major limitation because a previous study that applied the same diagnostic criteria for pulmonary TB showed a close correlation between case numbers in the NHIRD and those reported by the Taiwan Centers for Disease Control [TCDC] [29]. In addition, among all TB patients in 2009, the percentages of patients living with HIV were extremely similar between this study (0.55%) and the TCDC report (0.6%) [28].

Although the finding that cART was not associated with a low risk of TB recurrence was consistent with that of a previous report in Taiwan [30], this finding was different from that of a meta-analysis [26]. This is probably because the sample size was too small to demonstrate the protective effect of cART. In addition to the National Health Insurance system, rifabutin can be requested from the TCDC. Results of the analysis on non-adherent cases and duration of rifamycins (S1, S2 and S3 Tables) suggest this is a potential confounder in calculating the exposure duration of rifamycins and resulting in underestimate of the adherence to anti-TB treatment. Furthermore, without genotyping data, this study could not differentiate recurrence due to reactivation, which is the primary concern for extending anti-TB treatment, from relapse due to exogenous reinfection. This again biased the result toward null and caused underestimation of protection from extended anti-TB treatment.

In conclusion, we found that the TB recurrence rate within 2 years after completion of treatment has declined in HIV-infected patients in Taiwan since 2006, and the risk of TB recurrence can be reduced by extending anti-TB treatment to 9–12 months.

## Supporting Information

S1 Dataset (XLS)

S1 Fig (DOC)

S1 File (DOC)

S1 Table (DOC)

S2 Table (DOC)

S3 Table (DOC)

S4 Table (DOC)

S5 Table (DOC)
