# Supplementary material for: Nine- to Twelve-Month Anti-Tuberculosis Treatment Is Associated with a Lower Recurrence Rate than 6–9-Month Treatment in Human Immunodeficiency Virus-Infected Patients: A Retrospective Population-Based Cohort Study in Taiwan
Source: PLoS One. 2015 Dec 3;10(12):e0144136. doi: 10.1371/journal.pone.0144136 (PMC4669121; doi:10.1371/journal.pone.0144136)

**Fig. S1.** **Adjusted time-to-recurrence curves for the 449 patients with human immunodeficiency virus infection stratified by the timing of tuberculosis (TB) diagnosis (left panel) and duration of anti-TB treatment (right panel), plotted on the basis of regression estimates in the Cox model and average covariate values (average covariate method)**. DOTS, directly observed therapy, short course.


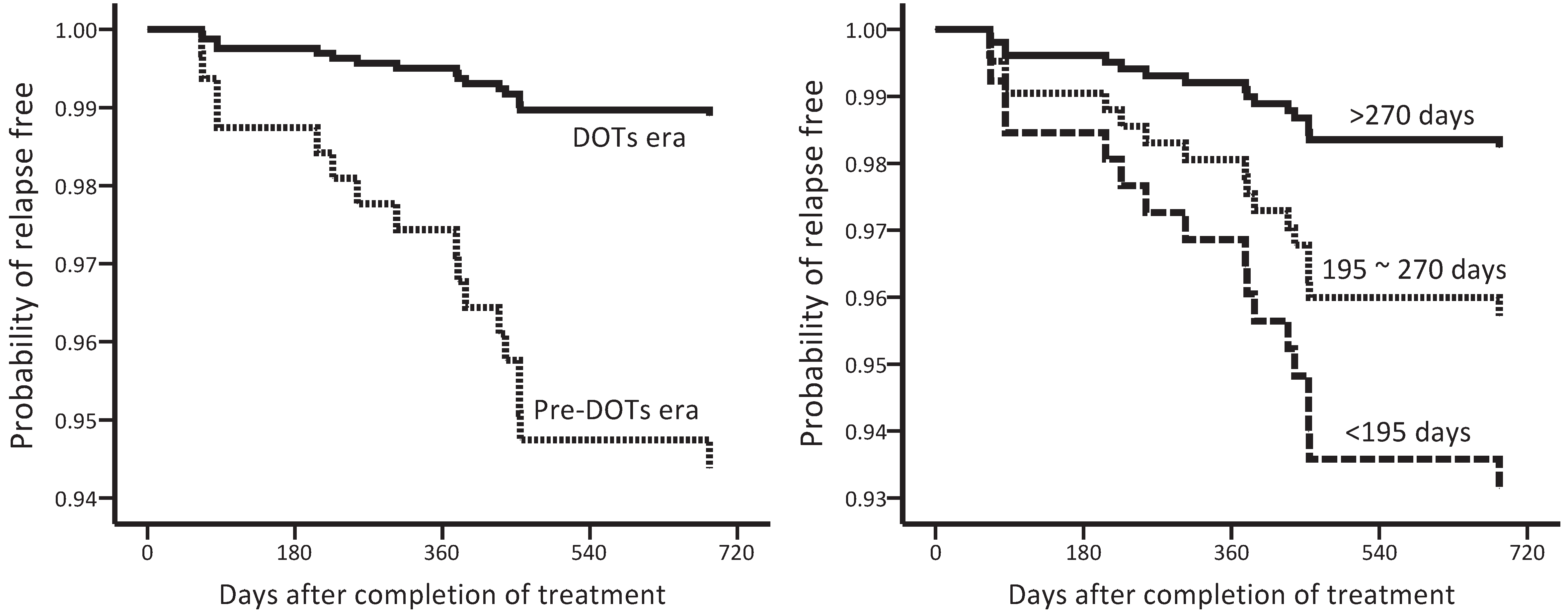

Supplement: S1 Fig — (DOC) [file pone.0144136.s002.doc]
