# Supplementary material for: Nine- to Twelve-Month Anti-Tuberculosis Treatment Is Associated with a Lower Recurrence Rate than 6–9-Month Treatment in Human Immunodeficiency Virus-Infected Patients: A Retrospective Population-Based Cohort Study in Taiwan
Source: PLoS One. 2015 Dec 3;10(12):e0144136. doi: 10.1371/journal.pone.0144136 (PMC4669121; doi:10.1371/journal.pone.0144136)
Supplement: S1 File — (DOC) [file pone.0144136.s003.doc]

**Supplementary File**

**Definition of Active Pulmonary Tuberculosis**

Patients with active pulmonary TB were identified as those having at least 2 ambulatory visits or 1 inpatient record with a compatible diagnosis and those who were prescribed at least 1 of 3 or more anti-TB drugs and at least 2 anti-TB drugs simultaneously for ≥120 days within a period of 180 days . Compatible diagnoses of active pulmonary TB were determined according to International Classification of Diseases, Ninth Revision, Clinical Modification (ICD-9-CM) codes 010–012 and 018 or A-codes A020 and A021. Among the TB patients, those with at least 2 ambulatory visits or 1 inpatient record with a diagnosis of CNS (ICD-9-CM code 013 or A-code A022) or musculoskeletal TB (ICD-9-CM code 015 or A-code A024) were excluded. Patients diagnosed with nontuberculous mycobacterial infection (ICD-9-CM code 031) during the final 2 months of anti-TB treatment were also excluded. The anti-TB drugs used were INH; rifamycins, including rifampin and rifabutin; EMB; PZA; and second-line drugs, including prothionamide, terizidone, streptomycin, kanamycin, cycloserine, aminosalicylic acid, and fluoroquinolones. Data on individual anti-TB agents extracted from the claims data were converted to the DDDs, and the agents were grouped according to their pharmacologic categories . The prescriptions of patients with end-stage renal disease were adjusted according to treatment guidelines .

**Definition of Human Immunodeficiency Virus Infection and Combination Antiretroviral Therapy**

The ambulatory care and inpatient discharge records were analyzed to identify any patient having an outpatient or hospitalization event with the diagnosis of HIV infection (ICD-9-CM codes 042–044 and V08). TB patients who had a diagnostic code of HIV infection within 3 years were considered to have HIV coinfection. Combination antiretroviral therapy (cART), which became available in Taiwan in early 1997, was defined as the simultaneous use of 2 NRTIs and an NNRTI or a PI . All antiretroviral regimens and monitoring of the plasma HIV RNA load and CD4 count are provided free of charge at designated hospitals throughout Taiwan. According to the national treatment guidelines, treatment with 2 NRTIs and efavirenz is the recommended regimen for patients receiving rifampin-containing anti-TB treatment; for those intolerant of efavirenz or infected with HIV-1 harboring resistance mutations for NNRTI, 2 NRTIs and a boosted PI are used in combination with rifabutin-containing anti-TB treatment . The DDDs of patients simultaneously prescribed efavirenz or ritonavir were adjusted accordingly .

**REFERENCES**
