# Supplementary material for: Nine- to Twelve-Month Anti-Tuberculosis Treatment Is Associated with a Lower Recurrence Rate than 6–9-Month Treatment in Human Immunodeficiency Virus-Infected Patients: A Retrospective Population-Based Cohort Study in Taiwan
Source: PLoS One. 2015 Dec 3;10(12):e0144136. doi: 10.1371/journal.pone.0144136 (PMC4669121; doi:10.1371/journal.pone.0144136)
Supplement: S1 Table — (DOC) [file pone.0144136.s004.doc]

**Supplementary Table 1. The exposure duration of each anti-tuberculosis drug according to the adherence on anti-tuberculosis treatment**

|  | **Intensive Phase** | | | |  | **6-Month** | | | |
| --- | --- | --- | --- | --- | --- | --- | --- | --- | --- |
|  | INH | Rifamycin | EMB | PZA |  | INH | Rifamycin | EMB | PZA |
| Adherent* (n=137) | 59.3 ± 1.6 | 57.3 ± 3.3 | 56.6 ± 2.9 | 57.0 ± 3.3 |  | 175.5 ± 7.0 | 171.2 ± 9.6 | 154.1 ± 29.8 | 95.5 ± 44.0 |
| Non-adherent (n=374) | 42.0 ± 21.1 | 43.2 ± 16.3 | 48.5 ± 12.4 | 36.1 ± 20.2 |  | 125.2 ± 63.1 | 130.9 ± 48.2 | 142.2 ± 41.8 | 75.7 ± 53.6 |
| *p*-value^#^ | <0.001 | <0.001 | <0.001 | <0.001 |  | <0.001 | <0.001 | <0.001 | <0.001 |

INH, isoniazid; EMB, ethambutol; PZA, pyrazinamide

Data were presented as mean ± SD unless otherwise mentioned

* A case was considered to be adherent to anti-tuberculosis treatment If receiving INH, a rifamycin, EMB, and PZA for >48 days in the first 2 months and INH and a rifamycin for >144 days in the first 6 months of treatment.

^#^ calculated by using independent-sample *t*-test
