# Supplementary material for: Nine- to Twelve-Month Anti-Tuberculosis Treatment Is Associated with a Lower Recurrence Rate than 6–9-Month Treatment in Human Immunodeficiency Virus-Infected Patients: A Retrospective Population-Based Cohort Study in Taiwan
Source: PLoS One. 2015 Dec 3;10(12):e0144136. doi: 10.1371/journal.pone.0144136 (PMC4669121; doi:10.1371/journal.pone.0144136)
Supplement: S2 Table — (DOC) [file pone.0144136.s005.doc]

**Supplementary Table 2. The exposure duration of rifamycin and isoniazid according to the numbers of reasons for non-adherence.** Overall, there were six reasons for non-adherence, i.e., (1) duration of isoniazid ≤48 days within first 2 months; (2) duration of rifamycin ≤48 days within first 2 months; (3) duration of ethambutol ≤48 days within first 2 months; (4) duration of pyrazinamide ≤48 days within first 2 months; (5) duration of isoniazid ≤144 days within 6 months; and (6) duration of rifamycin ≤144 days within 6 months.

| **No. of reasons for non-adherence** | **Rifamycin** | | | |  | **Isoniazid** | | | |
| --- | --- | --- | --- | --- | --- | --- | --- | --- | --- |
|  | ≤48 days within 2M | ≤144 days within 6M | Either one | Both |  | ≤48 days within 2M | ≤144 days within 6M | Either one | Both |
| One (n=89) | 3 | 7 | 10 | 0 |  | 2 | 9 | 11 | 0 |
| Two (n=82) | 31 | 39 | 46 | 24 |  | 24 | 32 | 36 | 20 |
| Three (n=72) | 37 | 47 | 56 | 28 |  | 34 | 35 | 47 | 22 |
| Four (n=62) | 53 | 39 | 61 | 31 |  | 45 | 30 | 52 | 23 |
| Five (n=42) | 42 | 35 | 42 | 35 |  | 39 | 18 | 42 | 15 |
| Six (n=27) | 27 | 27 | 27 | 27 |  | 27 | 27 | 27 | 27 |

Data were number of cases
