# Supplementary material for: Nine- to Twelve-Month Anti-Tuberculosis Treatment Is Associated with a Lower Recurrence Rate than 6–9-Month Treatment in Human Immunodeficiency Virus-Infected Patients: A Retrospective Population-Based Cohort Study in Taiwan
Source: PLoS One. 2015 Dec 3;10(12):e0144136. doi: 10.1371/journal.pone.0144136 (PMC4669121; doi:10.1371/journal.pone.0144136)
Supplement: S3 Table — (DOC) [file pone.0144136.s006.doc]

**Supplementary Table 3. The exposure duration of each anti-tuberculosis drug according to the status of anti-HIV therapy**

|  | Intensive Phase | | | |  | 6-Month | | | |
| --- | --- | --- | --- | --- | --- | --- | --- | --- | --- |
|  | INH | Rifamycin | EMB | PZA |  | INH | Rifamycin | EMB | PZA |
| No Tx (n=201) | 46.2 ± 20.5 | 49.4 ± 13.2 | 50.4 ± 12.3 | 42.3 ± 20.3 |  | 135.5 ± 60.2 | 148.6 ± 37.3 | 143.3 ± 40.3 | 75.9 ± 49.5 |
| Not cART (n=122) | 48.4 ± 17.9 | 49.5 ± 13.1 | 51.7 ± 10.3 | 43 ± 18.9 |  | 149 ± 52.5 | 150 ± 41.4 | 146.8 ± 39.7 | 82 ± 53.6 |
| cART (n=185) | 45.8 ± 19.8 | 42.6 ± 17.9 | 50.2 ± 10.9 | 40 ± 19.5 |  | 134.8 ± 60.2 | 128.4 ± 52.3 | 146.5 ± 38.1 | 85.6 ± 53.3 |
| *p*-value* | 0.499 | **<0.001** | 0.478 | 0.349 |  | 0.074 | **<0.001** | 0.654 | 0.186 |

INH, isoniazid; cART, combined antiretroviral therapy; EMB, ethambutol; PZA, pyrazinamide; Tx, treatment

Data were presented as mean ± SD unless otherwise mentioned

*calculated by using one-way analysis of variance
