# Supplementary material for: Nine- to Twelve-Month Anti-Tuberculosis Treatment Is Associated with a Lower Recurrence Rate than 6–9-Month Treatment in Human Immunodeficiency Virus-Infected Patients: A Retrospective Population-Based Cohort Study in Taiwan
Source: PLoS One. 2015 Dec 3;10(12):e0144136. doi: 10.1371/journal.pone.0144136 (PMC4669121; doi:10.1371/journal.pone.0144136)
Supplement: S4 Table — (DOC) [file pone.0144136.s007.doc]

**Supplementary Table 1. Clinical characteristics of the 499 patients with human immunodeficiency virus infection**

|  | <195 days  n=66 | 195~270 days  n=155 | >270 days  n=228 |
| --- | --- | --- | --- |
| Age (year) | 39.4 ± 11.8 | 41.6 ± 14.3 | 40.7 ± 12.1 |
| Age >49 | 11 (16.7%) | 39 (25.2%) | 52 (22.8%) |
| Male | 62 (93.9%) | 148 (95.5%) | 208 (91.2%) |
| Timing of TB diagnosis |  |  |  |
| Pre-DOTS era | 36 (54.5%) | 89 (57.4%) | 138 (60.5%) |
| DOTS era | 30 (45.5%) | 66 (42.6%) | 90 (39.5%) |
| Comorbidity | 6 (9.1%) | 20 (12.9%) | 17 (7.5%) |
| Diabetic mellitus | 3 (4.5%) | 13 (8.4%) | 15 (6.6%) |
| Chronic obstructive pulmonary disease | 2 (3.0%) | 3 (1.9%) | 2 (0.9%) |
| Malignancy* | 2 (3.0%) | 5 (3.2%) | 0 (0.0%) |
| End-stage renal disease | 0 (0.0%) | 1 (0.6%) | 1 (0.4%) |
| Low income | 1 (1.5%) | 3 (1.9%) | 5 (2.2%) |
| Duration of anti-TB Tx (days)** | 182.2 ± 8.6 | 234.7 ± 24.0 | 318.9 ± 34.2 |
| Total duration of isoniazid administration** | 153.5 ± 46.9 | 177.3 ± 79.5 | 236.6 ± 109.6 |
| ≥240 days** | 0 (0.0%) | 31 (20.0%) | 160 (70.2%) |
| Total duration of rifamycin administration** | 151.1 ± 42.3 | 179.1 ± 62.4 | 234.2 ± 94.1 |
| ≥240 days** | 0 (0.0%) | 21 (13.5%) | 140 (61.4%) |
| Total duration of ethambutol administration** | 143.7 ± 40.4 | 180.0 ± 59.6 | 251.8 ± 78.6 |
| ≥240 days** | 0 (0.0%) | 20 (12.9%) | 165 (72.4%) |
| Total duration of pyrazinamide administration* | 84.0 ± 48.6 | 86.7 ± 68.5 | 107.5 ± 90.5 |
| ≥60 days | 49 (74.2%) | 92 (59.4%) | 151 (66.2%) |
| ≥90 days | 25 (37.9%) | 57 (36.8%) | 97 (42.5%) |
| 80% consistence with standards*^#^ | 26 (39.4%) | 35 (22.6%) | 45 (19.7%) |
| Intensive phase (initial 60 days) |  |  |  |
| No. of days covered by isoniazid* | 51.9 ± 16.0 | 45.6 ± 19.7 | 44.7 ± 20.6 |
| No. of days covered by rifamycin* | 51.6 ± 11.2 | 45.6 ± 15.4 | 44.5 ± 16.9 |
| No. of days covered by ethambutol | 52.9 ± 9.1 | 50.3 ± 10.5 | 49.9 ± 12.1 |
| No. of days covered by pyrazinamide* | 46.3 ± 18.3 | 39.0 ± 20.7 | 40.2 ± 19.7 |
| Anti-HIV therapy during anti-TB Tx** |  |  |  |
| cART | 19 (28.8%) | 55 (35.5%) | 111 (48.7%) |
| Yes, but not cART | 17 (25.8%) | 38 (24.5%) | 67 (29.4%) |
| No | 30 (45.5%) | 62 (40.0%) | 50 (21.9%) |
| 2-year recurrence after anti-TB Tx* | 5 (7.6%) | 8 (5.2%) | 4 (1.8%) |

cART, combined antiretroviral therapy; DOTS, directly observed therapy, short course; Tx, treatment

Data were presented either number (%) or mean ± SD

**p* < 0.05 and ***p* < 0.001 between the 3 groups with different durations of anti-TB treatment according to one-way analysis of variance for continuous variables or the chi-square test for categorical variables.

^#^receiving isoniazid, rifamycin, ethambutol, and pyrazinamide for >48 days in the first 2 months, and isoniazid and rifamycin for >144 days in the first 6 months of anti-TB treatment.
