# Supplementary material for: Nine- to Twelve-Month Anti-Tuberculosis Treatment Is Associated with a Lower Recurrence Rate than 6–9-Month Treatment in Human Immunodeficiency Virus-Infected Patients: A Retrospective Population-Based Cohort Study in Taiwan
Source: PLoS One. 2015 Dec 3;10(12):e0144136. doi: 10.1371/journal.pone.0144136 (PMC4669121; doi:10.1371/journal.pone.0144136)
Supplement: S5 Table — (DOC) [file pone.0144136.s008.doc]

**Supplementary Table 2. Determination of independent risk factors for tuberculosis (TB) recurrence within 2 years after completion of anti-TB treatment among the 449 patients selected for sensitivity analysis by using Cox proportional hazards regression analysis**

|  | *p* value | Hazard ratio | 95% CI | |
| --- | --- | --- | --- | --- |
|  |  |  | Lower | Upper |
| Timing of TB diagnosis: DOTS era *vs*. pre-DOTS era | 0.028 | 0.19 | 0.04 | 0.84 |
| Duration of anti-TB treatment: |  |  |  |  |
| >270 days vs. <195 days | 0.029 | 0.25 | 0.07 | 0.87 |
| 195–270 days vs. <195 days | 0.408 | 0.62 | 0.20 | 1.94 |

DOTS, directly observed therapy, short course

Patients fulfilling any of the following criteria were selected for sensitivity analysis: (1) those who had received anti-human immunodeficiency virus (HIV) medication; (2) those who had at least 1 admission with the discharge diagnosis of HIV; and (3) those who had 2 outpatient visits with the diagnosis of HIV within 360 calendar days.
